# Corin Is Downregulated in Renal Ischemia/Reperfusion Injury and Is Associated with Delayed Graft Function after Kidney Transplantation

**DOI:** 10.1155/2019/9429323

**Published:** 2019-01-14

**Authors:** Xinyi Hu, Ming Su, Jun Lin, Lei Zhang, Wen Sun, Jian Zhang, Ye Tian, Wei Qiu

**Affiliations:** ^1^Department of Urology, Beijing Friendship Hospital, Capital Medical University, Beijing, China; ^2^Department of Clinical Laboratory, Peking University People's Hospital, Beijing, China

## Abstract

Renal ischemia/reperfusion (IR) injury is one of the most important risk factors for the occurrence of delayed graft function (DGF) after kidney transplantation; however, its mechanism remains not fully understood. In the present study, we screened differentially expressed genes in a murine model of renal IR injury by using high-throughput assays. We identified *Corin* as one of the most significantly downregulated genes among 2218 differentially expressed genes (≥2-fold, *P* < 0.05). By using a real-time qPCR assay, we observed that the expression of renal *Corin* in IR-injured mice was reduced to 11.5% of the sham-operated mice and that the protein level of renal Corin in IR-injured mice was also downregulated. Interestingly, renal IR injury in mice induced the downregulation of Corin in heart tissues, suggesting that the overall synthesis of Corin may be suppressed. We recruited 11 recipients complicated with DGF and 16 without DGF, and plasma Corin concentrations were determined by ELISA. We observed that the plasma Corin levels were indeed reduced in recipients complicated with DGF (0.98 vs. 1.95 ng/ml, *P* < 0.05). These findings demonstrate that Corin may be a potential biomarker of DGF after kidney transplantation and may participate in the regulation of renal IR injury.

## 1. Introduction

With extended criteria for deceased kidney donors, ischemia/reperfusion (IR) injury is becoming a more frequent event during kidney transplantation. IR injury causes high rates of delayed graft function (DGF) or primary graft nonfunction (PGN), which significantly influences the outcome of kidney transplantation. The IR injury initially induces oxidative stress, reactive oxygen species (ROS) generation, and acute inflammation and damages the tubular cells and endothelial cells, causing acute kidney injury (AKI) [1]. Hypoxia induces the release of several factors such as transforming growth factor-*β*1, evoking interstitial fibrosis, and aggravating DGF [2].

Atrial natriuretic peptide (ANP), a peptide hormone mostly produced in the atria, is a potent endocrine factor regulating the extracellular fluid volume and blood pressure [3]. ANP acts on the kidney and promotes natriuresis. In experimental AKI models, exogenous infusion of ANP protects against kidney injury [4]. Thus, ANP may act as a potential therapeutic candidate for AKI in kidney transplantation. The generation of mature ANP is dependent on a serine protease named as Corin. This enzyme is abundantly expressed in the heart, mediating the cleavage of pro-ANP to its activated form. Recent studies have indicated that Corin is also present in renal tissue [5], and the decrease in renal Corin is associated with chronic kidney disease [6]. Another group reported that proinflammation factors such as interleukin-1*β* (IL-1*β*) and tumor necrosis factor-*α* (TNF-*α*) upregulate Corin expression in patients with primary proteinuric kidney diseases [7]. However, the role of Corin in renal IR injury remains unclear. In the present study, we screened for differentially expressed genes in renal IR-injured mice using a high-throughput assay. We found that *Corin* was significantly downregulated in renal IR injury, indicating a probable association between Corin and renal IR injury.

## 2. Materials and Methods

### 2.1. Ethical Statements

The studies that related to patients were approved by the Ethics Committee of Beijing Friendship Hospital, Capital Medical University, according to the Declaration of Helsinki, and informed consent was obtained from all patients. The procedure of the study followed the Declaration of Istanbul, 2008, on Organ Trafficking and Transplant Tourism, endorsed by The Transplantation Society (TTS) and the International Society of Nephrology (ISN). The studies related to animals were approved by the Beijing Friendship Hospital Animal Care and Use Committee.

### 2.2. Recipients

A total of 27 recipient patients that had undergone kidney transplantation in Beijing Friendship Hospital, Capital Medical University, were recruited in the study. All of the transplantation was performed from deceased donors. The serum samples from all the recipients were collected at 24 h post operation. The daily serum creatinine (Cr) levels of all recipients were monitored. A diagnosis of DGF was defined by the failure of serum creatinine levels to decrease after transplantation; otherwise, the recipients were enrolled into the immediate graft function (IGF) group.

### 2.3. Animal Studies

The IR injury model was performed as previously described [8, 9]. In brief, 8-week-old male C57/BL6 mice were anesthetized with pentobarbital sodium (0.05 mg/g body weight) through intraperitoneal (IP) injection and randomly divided into injury and sham-operated groups (*n* = 5 per group). For renal IR injury, the mice were subjected to 45 min ischemia by bilaterally clamping the renal artery. After 24 h of reperfusion, the mice were killed, and the serum, kidney, and heart samples were collected and immediately frozen at -80°C or fixed in 4% paraformaldehyde solution for histological analysis.

### 2.4. Cell Culture and Treatment

Human kidney 2 (HK2) cells were obtained from the National Infrastructure of Cell Line Resource (Beijing, China). The cells were cultured in DMEM/F12 containing 10% fetal bovine serum (FBS) in an atmosphere of 5% CO_2_ at 37°C.

The cells were subjected to oxygen-glucose deprivation/reoxygenation (OGD/R) injury. In brief, HK2 cells were washed with PBS and maintained in glucose-free DMEM (Thermo Fisher Scientific, Schuylerville, NY, USA). The cells were exposed to an atmosphere of 1% O_2_, 5% CO_2_, and 94% N_2_ at 37°C for 2 h. Then, the cells were transferred into a complete medium and cultured under routine conditions for 24 h. Normally cultured HK2 cells were used as the control. The conditioned medium was collected and centrifuged to obtain the cell-free supernatant for further measurement.

### 2.5. Enzyme-Linked Immunosorbent Assay

The secreted form of Corin was detected using a human Corin enzyme-linked immunosorbent assay (ELISA) kit (Cusabio, Wuhan, China). The procedure was performed according to the manufacturer's instruction. Briefly, 100 *μ*l of a cell-free medium or plasma sample was transferred to the Corin antibody-precoated 96-well plate and incubated at 37°C for 2 h. The supernatant was then replaced with 100 *μ*l of biotin-modified antibody and incubated at 37°C for 1 h. The wells were washed 3 times using a washing buffer, and 100 *μ*l of HRP-tagged avidin was loaded onto the plate and incubated at 37°C for 1 h. The supernatant was then discarded and washed 5 times. After that, 90 *μ*l of TMB substrate was added into the wells and maintained at 37°C for 30 min, and the reaction was stopped by adding 50 *μ*l of stop solution. The optical density (OD) was read with a microplate reader at 450 nm. A gradient dilution of Corin protein was used to construct the standard curve. The concentration of Corin in each well was calculated.

### 2.6. Microarray Assay

Total RNA from the mouse kidneys was extracted using a TRIzol reagent (Thermo Fisher Scientific). For screening the differentially expressed genes, 3 mice that had undergone IR injury and 3 sham-operated mice were included. The expression profiles were performed at the Shanghai Biotechnology Corporation (Shanghai, China) using an Affymetrix Mouse Genome 430 2.0 microarray (Thermo Fisher Scientific). The genes that exhibited at least a 2-fold difference in expression (*P* < 0.05) between groups were considered for further investigation.

### 2.7. RNA Analysis

Complementary DNAs (cDNAs) were synthesized with a cDNA Synthesis Kit (Takara, Dalian, China). Real-time quantitative PCR (qPCR) was performed with specific primers: mouse *Corin*: forward: 5′ATCAGCTGGGAGTCATCCCT3′, reverse: 5′GGAGTGCTCACACAGGAGTC3′; mouse *ANP*: forward: 5′AGTGCGGTGTCCAACACAG3′, reverse: 5′TGCTTCCTCAGTCTGCTCACTC3′; and mouse *BNP*: forward: 5′CTTTATCTGTCACCGCTGGGAG3′, reverse: 5′TTTGGGTGTTCTTTTGTGAGGC3′. *GAPDH* was used as an internal control: forward: 5′GGCATTGTGGAAGGGCTC3′ and reverse: 5′GGGGGTAGGAACACGGAAG3′. The relative expression was calculated using the 2^-ΔΔCt^ method.

### 2.8. Histological Analysis

Kidneys from each group were fixed in 4% paraformaldehyde solution, embedded in paraffin, and cut into 4 *μ*m sections. Hematoxylin and eosin staining was used for pathological analysis. The endogenous Corin was stained with a rabbit anti-Corin (sc-67178, Santa Cruz, Dallas, TX, USA) primary antibody by using immunohistochemistry (IHC) staining assay.

### 2.9. Protein Analysis

Total protein from the tissues was lysed, quantified, and denatured, and 50 to 150 *μ*g of protein was loaded for SDS polyacrylamide gel electrophoretic separation. The proteins were blotted onto a nitrocellulose membrane. Then, the membrane was soaked in 5% nonfat milk at room temperature for 1 h. The membrane was incubated with rabbit anti-Corin (Santa Cruz) or rabbit anti-*β*-actin (Cell Signaling Technology, Beverly, MA, USA) primary antibodies at 4°C overnight. After the incubation, the membrane was washed with TBST solution four times, and the membrane was incubated with an HRP-conjugated goat anti-rabbit secondary antibody for 1 h at room temperature. The membrane was washed again with TBST solution 4 times, and the specific bands were detected using SuperSignal West Femto Maximum Sensitivity Substrate (Pierce, Rockford, IL, USA), and *β*-actin was employed as a loading control for normalization.

### 2.10. Statistical Analysis

The statistical significance was analyzed using two-tailed Student's *t*-test or Pearson's *χ* ^2^ test with SPSS 19.0 software (SPSS Inc., Chicago, IL, USA). *P* < 0.05 was considered statistically significant. The data are expressed as mean ± SEM except for special instructions.

## 3. Results

### 3.1. The Kidney Corin-ANP System Is Altered in Ischemia/Reperfusion Injury

**Figure 1:**
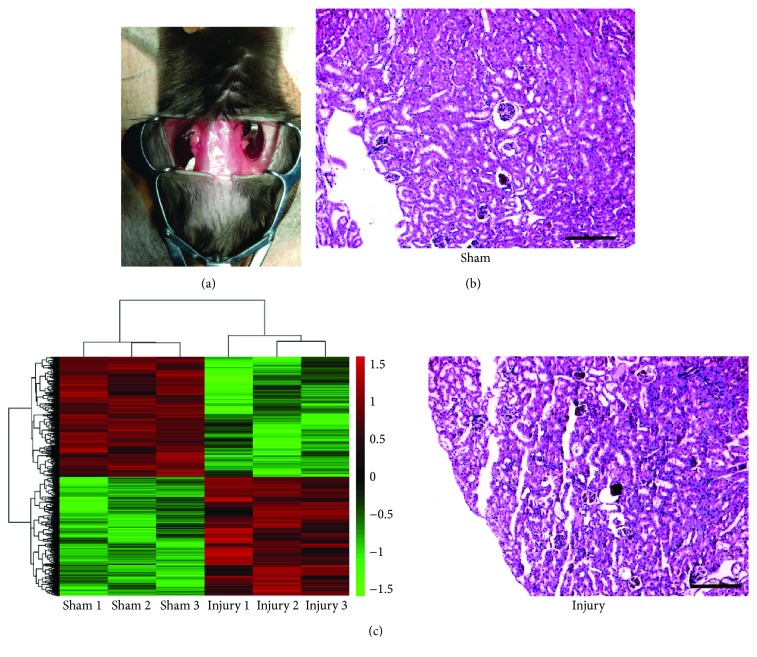
Screening for the differentially expressed genes in renal IR injury. (a) Representative image of the renal IR injury operation in mice. (b) Pathological changes of the IR injury in the kidneys. The sections were stained with hematoxylin and eosin (H&E), and representative images are shown, scale bars: 200 *μ*m. (c) Heat map diagram of differentially expressed genes from 3 IR-injured mice and 3 sham-operated mice. Red in the color bar shows higher expression and green is lower expression.

**Figure 2:**
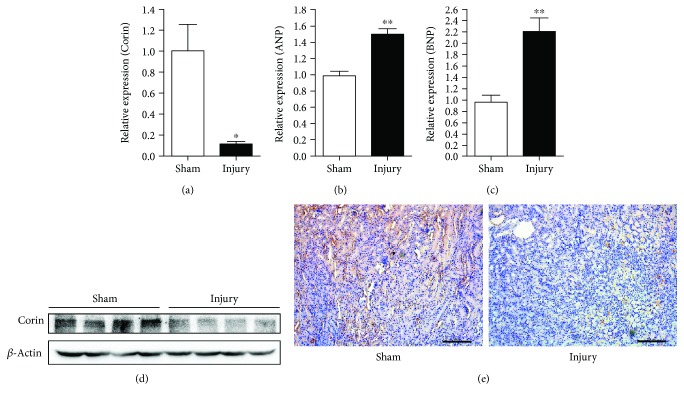
Renal Corin is downregulated in IR injury. (a-c) The expression of *Corin* (a), *ANP* (b), and *BNP* (c) was measured using a real-time qPCR assay. *GAPDH* was used as an internal control, and the data are normalized using a 2^-ΔΔCt^ assay. Data are expressed as mean ± SEM, ^∗^ *P* < 0.05 and ^∗∗^ *P* < 0.01 vs. the sham-operated group. (d) The protein level of Corin was detected by western blotting. *β*-Actin was employed as an internal control, *n* = 4 per group. (e) Immunohistochemistry staining against Corin was performed in kidney tissues. Representative images are shown, scale bars: 200 *μ*m.

In order to screen for differentially expressed genes in IR-injured kidneys, we initially constructed the renal IR injury mouse model by bilateral clamping of the renal artery and reperfusion (Figure 1(a)). Histological analysis verified the injury of the kidneys (Figure 1(b)). The expression profiles in IR-injured or sham-treated kidneys were measured using a microarray assay. We found 2218 differentially expressed genes (≥2-fold, *P* < 0.05), including 1103 upregulated and 1115 downregulated genes (Figure 1(c) and Supplementary Material 1). In these genes, we identified that *Corin* ranked as one of the most significant downregulated genes. A real-time qPCR assay was performed to verify these changes. As is shown in Figure 2(a), the expression of renal *Corin* was downregulated to 11.5% of the sham-operated mice. Consistently, the expression of Corin at the protein level was significantly downregulated (Figure 2(b)). By using IHC assays, we observed that Corin was modestly expressed in normal kidney tubules, whereas IR injury suppressed its expression (Figure 2(c)).

### 3.2. Renal IR Injury Downregulates Cardiac Corin Expression

**Figure 3:**
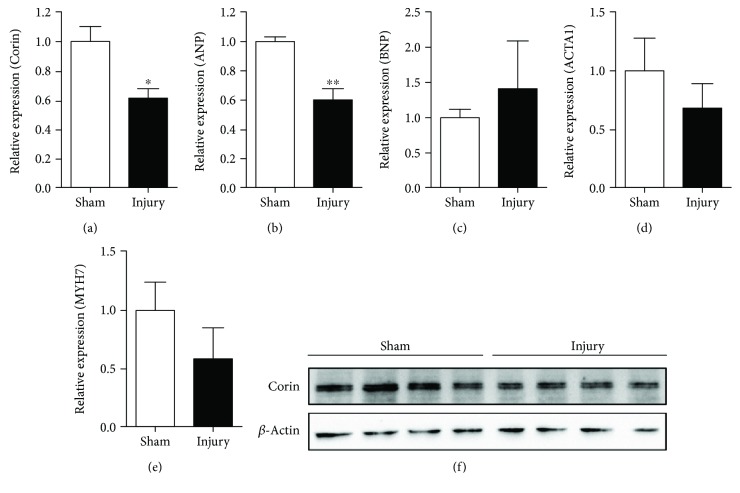
Renal IR injury suppresses the expression of cardiac Corin. Cardiac *Corin* (a) and cardiac biomarkers *ANP* (b), *BNP* (c), *ACTA1* (d), and *MYH7* (e) were detected using a real-time qPCR assay. *GAPDH* was used as an internal control, and the data are normalized using a 2^-ΔΔCt^ assay. Data are expressed as mean ± SEM, ^∗^ *P* < 0.05 and ^∗∗^ *P* < 0.01 vs. the sham-operated group. (f) Cardiac Corin protein was detected using western blotting. *β*-Actin was employed as an internal control, *n* = 4 per group.

Corin is the only enzyme mediating the cleavage of pre-ANP into mature ANP. Because the Corin-ANP system is mainly synthesized in heart tissues, we compared the changes in the Corin-ANP system after renal IR injury. Interestingly, we observed that the expression of *Corin* and *ANP* was downregulated in renal IR-injured mice (Figures 3(a), 3(b), and 3(f)). However, other cardiac remodeling markers, such as *BNP*, *ACTA1*, and *MYH7*, were all comparable in renal IR-injured mice (Figures 3(c)–3(e)). These data indicated that renal IR might distantly influence the cardiac Corin-ANP system, although the mechanism remains to be investigated.

### 3.3. Oxygen-Glucose Deprivation/Reoxygenation Downregulates Corin in Renal Tubular Epithelial Cells

**Figure 4:**
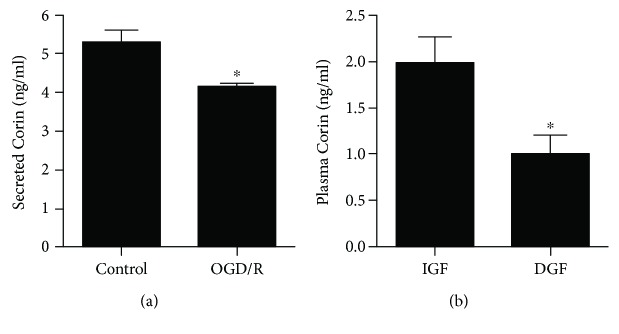
Corin is related with renal IR injury-induced delayed graft function of kidney transplantation. (a) HK2 cells were treated with OGD/R. The secreted Corin in the conditioned medium was determined using an ELISA. Data are expressed as mean ± SEM, ^∗^ *P* < 0.05 vs. the control group. (b) The plasma Corin of the recipients with DGF (delayed graft function, *n* = 11) or IGF (immediate graft function, *n* = 16) was determined using an ELISA. Data are expressed as mean ± SEM, ^∗^ *P* < 0.05 vs. IGF recipients.

To explore whether the downregulation of Corin is a direct effect of IR injury, OGD/R experiments were performed to mimic IR injury in vitro. HK2 cells were subjected to OGD for 1 h and reoxygenated for 24 h. By using an ELISA, we found that the secreted Corin in the medium was significantly reduced (Figure 4(a)), indicating that OGD/R treatment suppresses the secretion of Corin in renal tubular epithelial cells.

### 3.4. The Plasma Corin Level Is Negatively Associated with DGF after Kidney Transplantation

Since OGD/R upregulates the secreted Corin in renal tubular epithelial cells, we hypothesized that plasma Corin might be associated with renal IR injury-induced DGF in kidney transplantation. To verify our hypothesis, we recruited 27 recipients who had undergone kidney transplantation, including 11 recipients complicated with DGF and 16 recipients without DGF (enrolled in the IGF group). The clinical characteristics are shown in Table 1 and Supplementary Material 2. Clinical parameters such as recipient age, gender, body weight, dialysis vintage, comorbidities, donor age, donor gender, and cold ischemic time were all comparable between DGF and IGF. The plasma Corin was determined using an ELISA. We observed that the plasma Corin level was significantly decreased in recipients with DGF compared with the IGF recipients (0.98 vs. 1.95 ng/ml, respectively, *P* < 0.05). These data supported that plasma Corin is negatively associated with the occurrence of DGF after kidney transplantation, which highlights that circulatory Corin might be a potential biomarker for DGF after transplantation. Besides, we observed that the plasma N-terminal prohormone of BNP (NT-proBNP) after transplantation in DGF recipients was higher than that in IGF recipients; however, it was comparable before transplantation (Table 1).

The Corin-ANP system plays a crucial role in maintaining blood pressure and salt-water balance. Corin was primarily found in the atrium; subsequently, it was also identified in many other organs like the uterus, skin, brain, and kidney [10, 11]. The kidney-originated Corin was then reported to be associated with chronic kidney diseases [6, 12]; however, its function in the kidney remains unclear. In the present study, we observed that the plasma Corin level was associated with the occurrence of DGF after kidney transplantation, which indicated that Corin might be a biomarker for the renal IR injury-induced DGF.

## 4. Discussion

The principal role of Corin is dependent on its serine protease activity on the cleavage of pro-ANP. The generation of the mature form of ANP requires two main steps. The primary synthesized ANP, named as prepro-ANP, is initially processed by removing the signal peptide to generate a pro-ANP and subsequently cleaved by Corin into mature ANP [5, 13]. Corin also attenuates the maturation of BNP, although this process is not fully required. ANP is a well-known peptide hormone maintaining blood pressure and salt-water balance [14]. The receptor of ANP, named as natriuretic peptide receptor A (NPR-A), is abundantly expressed in the kidney tissues [15]. ANP binds to NPR-A and promotes renal sodium excretion. ANP is a potent vasodilator and antagonizes the renin–angiotensin–aldosterone system [16]. Corin is known as the only enzyme mediating the pro-ANP conversion, whereas knocking it out in mice fails to generate mature ANP, exhibiting salt-sensitive hypertension and cardiac hypertrophy [17, 18]. The Corin protease is present as the transmembrane form and could be cleaved to release into the circulation [19, 20]. The decreased circulatory levels of Corin could be detected in some cardiovascular diseases such as acute myocardial infarction, heart failure, and stroke [21–27]. Increased serum levels were found in hypertension, obesity, dyslipidemia, hyperglycemia, and atrial fibrillation [28–32]. In the present study, we observed that plasma Corin was negatively associated with the onset of DGF after kidney transplantation. However, both the renal and cardiac *Corin* may contribute to the decrease of Corin during renal IR injury.

Renal Corin is mostly presented in the epithelial cell surface of renal tubules and could be secreted into the urine, which is distinguished from the circulatory Corin [6]. Corin expression is mostly controlled by GATA-4 [33] and negatively regulated by IRE1 [34]. The IRE pathway is associated with the decrease in Corin in the failing heart [34]. In kidney tissues, Corin and ANP are coexpressed [35]; however, their functions remain unclear, despite the kidney being the main target organ of ANP. One study indicated that the urinary Corin level is reduced in chronic kidney disease and is associated with the severity [6]. Renal Corin and both the mRNA and protein levels were found to be negatively related with chronic kidney disease, supporting that the decrease of renal Corin might be a novel biomarker of kidney injury. Another group reported that IL-1*β* increases urinary Corin levels in primary proteinuric kidney diseases [7]. However, none of these studies separately analyzed the roles of renal and cardiac Corin. In our study, we observed a significant decrease in renal Corin in IR-injured mice. Interestingly, the expression of both renal ANP and BNP was upregulated; however, the mechanism for this currently remains unclear. Vinot et al. reported that the plasma ANP showed similar concentration in recipients of DGF and IGF [36]. Although we did not detect the plasma ANP in the subjects, our data indicated that the plasma NT-proBNP was indeed higher in DGF compared with IGF recipients. These changes might be a response for indicating the recovery of renal function after kidney transplantation.

In the present study, we investigated the response of the cardiac Corin-ANP system as well as BNP and other cardiac remodeling markers in IR-injured mice. Our data indicated that cardiac Corin was also downregulated in renal IR injury. In contrast with the kidney, cardiac ANP was downregulated, whereas the expression of cardiac BNP was comparable. We postulate that the injured kidney might secrete some factors and thus distantly influence the cardiac Corin-ANP system. However, these findings remain to be investigated in future studies.

## 5. Conclusions

IR injury is one of the most crucial but inevitable risks for the occurrence of DGF and PGN after kidney transplantation. Prolonged dialysis is required in patients complicated with DGF or PGN, causing a heavy burden to the recipients both mentally and physically. Our data revealed that renal IR injury reduced both cardiac and renal Corin, and more importantly, plasma Corin was downregulated in kidney transplantation recipients complicated with DGF. Therefore, Corin might be a potential biomarker that is associated with DGF of kidney transplantation.

## Figures and Tables

**Table 1:** Clinical characteristics of the subjects.

	DGF (*n* = 11)	IGF (*n* = 16)	*P* value
Recipient age (year)^§^	46.7 ± 11.4	48.6 ± 13.4	0.640
Recipient male (no. (%))	7 (63.6)	11 (68.8)	1.000^∗^
Weight (kg)^§^	66.2 ± 9.0	66.9 ± 9.97	0.855
Dialysis vintage (month)	12 (19.5)^$^	7.5 (22.3)^$^	0.412^#^
Hypertension (no. (%))	9 (81.8)	9 (56.3)	0.231^∗^
Diabetes (no. (%))	2 (18.2)	2 (12.5)	1.000^∗^
Donor age (year)^§^	48.7 ± 3.64	48.0 ± 8.86	0.800
Donor male (no. (%))	11 (100)	14 (87.5)	0.499^∗^
Cold ischemic time (hour)^§^	6.18 ± 1.66	5.75 ± 1.53	0.493
NT-proBNP before transplantation (ng/l)	3540 (7655)^$^	2095 (2752)^$^	0.120^#^
NT-proBNP after transplantation (ng/l)	3410 (4020)^$^	1420 (2107)^$^	0.030^#^

^§^Data are expressed as mean ± STD. ^∗^Fisher's exact test; ^$^median (interquartile range); ^#^Mann-Whitney *U* test.

## Data Availability

The original data used to support the findings of this study are included within the supplementary information file. The rest of all the data used to support the findings of this study are included within the article.
